# Targeted NGS, array-CGH, and patient-derived tumor xenografts for precision medicine in advanced breast cancer: a single-center prospective study

**DOI:** 10.18632/oncotarget.12714

**Published:** 2016-10-18

**Authors:** Anthony Gonçalves, François Bertucci, Arnaud Guille, Severine Garnier, José Adelaide, Nadine Carbuccia, Oliver Cabaud, Pascal Finetti, Serge Brunelle, Gilles Piana, Jeanne Tomassin-Piana, Maria Paciencia, Eric Lambaudie, Cornel Popovici, Renaud Sabatier, Carole Tarpin, Magali Provansal, Jean-Marc Extra, François Eisinger, Hagay Sobol, Patrice Viens, Marc Lopez, Christophe Ginestier, Emmanuelle Charafe-Jauffret, Max Chaffanet, Daniel Birnbaum

**Affiliations:** ^1^ Department of Medical Oncology, Institut Paoli-Calmettes, Marseille, France; ^2^ Aix Marseille Univ, CNRS U7258, INSERM U1068, Institut Paoli-Calmettes, CRCM, Marseille, France; ^3^ Department of Molecular Oncology, Institut Paoli-Calmettes, Marseille, France; ^4^ Department of Imaging, Institut Paoli-Calmettes, Marseille, France; ^5^ Department of Biopathology, Institut Paoli-Calmettes, Marseille, France; ^6^ Department of Surgical Oncology, Institut Paoli-Calmettes, Marseille, France; ^7^ Department of Oncogenetics, Institut Paoli-Calmettes, Marseille, France

**Keywords:** precision medicine, advanced breast cancer, NGS, CGH, patient-derived xenograft

## Abstract

**Background:**

Routine feasibility and clinical impact of genomics-based tumor profiling in advanced breast cancer (aBC) remains to be determined. We conducted a pilot study to evaluate whether precision medicine could be prospectively implemented for aBC patients in a single center and to examine whether patient-derived tumor xenografts (PDX) could be obtained in this population.

**Results:**

Thirty-four aBC patients were included. Actionable targets were found in 28 patients (82%). A targeted therapy could be proposed to 22 patients (64%), either through a clinical trial (n=15) and/or using already registered drugs (n=21). Ten patients (29%) eventually received targeted treatment, 2 of them deriving clinical benefit. Of 22 patients subjected to mouse implantation, 10 had successful xenografting (45%), mostly in triple-negative aBC.

**Methods:**

aBC patients accessible to tumor biopsy were prospectively enrolled at the Institut Paoli-Calmettes in the BC-BIO study (ClinicalTrials.gov, NCT01521676). Genomic profiling was established by whole-genome array comparative genomic hybridization (aCGH) and targeted next-generation sequencing (NGS) of 365 candidate cancer genes. For a subset of patients, a sample of fresh tumor was orthotopically implanted in humanized cleared fat pads of NSG mice for establishing PDX.

**Conclusions:**

Precision medicine can be implemented in a single center in the context of clinical practice and may allow genomic-driven treatment in approximately 30% of aBC patients. PDX may be obtained in a significant fraction of cases.

## INTRODUCTION

With more than 1.6 million of cases per year worldwide, breast cancer is the most common female cancer and the first cause of death by cancer in women [1]. In spite of major achievements in treating early-stage disease, a significant number of patients will be diagnosed with metastatic or unresectable locally-advanced disease (the so-called advanced-stage breast cancer, aBC), either *de novo* at initial presentation or during subsequent follow-up [2]. While the clinical outcome in this setting is highly variable, with some individuals experiencing long-term survival, most of aBC patients will die from the disease and their therapeutic management remains essentially palliative [3]. In the early 2000s, gene expression profiling studies have revealed some molecular basis of the clinical heterogeneity of breast cancer, identifying different transcriptional subtypes with distinct clinical pattern, survival outcome and therapeutic response [4]. More recently, comprehensive genomics-based analyses of large series of primary breast cancers using high-throughput next-generation sequencing (NGS) technologies have revealed an additional level of molecular diversity, with a large number of DNA mutations and structural alterations associated to previously described subtypes, some of which playing a driver role in oncogenesis [5, 6]. In precision cancer medicine, patients are stratified according to molecular characteristics of their tumors and specific therapeutics are selected according to corresponding expected efficacy [7]. This strategy was recently boosted by the rapid development and availability of novel technologies allowing probing tumor genome for DNA alterations, which could confer drug sensitivity to various targeted therapeutics. In breast cancer, precision medicine was first illustrated by endocrine and anti-HER2 treatments. Both strategies demonstrated exquisite antitumor activity in specific subsets of patients for whom the presence of the drug target was documented, namely in estrogen receptor (ER)-positive and HER2-overexpressing subtypes, respectively [8–11]. According to recent genotyping studies [5, 12, 13], previously known breast cancer subtypes could be further segmented in a large number of genetically-based subsets, many of which could be targeted by specific therapeutics. Recently, the multicentric, national, prospective SAFIR-01 trial provided the first proof of concept that genomic profiling, including whole-genome array-comparative genomic hybridization (aCGH) and limited Sanger-based DNA sequencing, could be implemented in aBC patients. However, the actual percentage of patients receiving effective targeted therapies was low, as well as the number of patients experiencing a clinical benefit [14]. In addition, the feasibility of such a program in a single-center and its actual impact on clinical management of patients has to be further evaluated.

Patient-derived tumor xenograft (PDX) models, in which fresh tumor samples are implanted directly into immune-compromised mice, recapitulate complexity and heterogeneity of the tumor of origin at cellular, molecular, genomic, and histological levels [15–17]. Thus, PDXs could be used as avatar mouse models to permit bench testing of various treatment strategies, including those proposed from genomic analysis. However, its implementation in clinical practice has not been determined in aBC patients.

Herein, we report a pilot prospective study enrolling aBC patients, in which we have evaluated a precision medicine-based strategy, including targeted NGS, aCGH, and PDX analyses. The primary objective was to prospectively determine the number of patients with actionable molecular alterations. The secondary objectives were to evaluate the number of patients who might be candidate to molecular-driven clinical trials or off-trial targeted treatments and the antitumor activity of delivered treatments. Other aims included an approximation of the percentage of PDX that could be derived from tumor biopsy obtained in aBC patients as well as exploratory correlations between identified molecular alterations and clinico-pathological features.

## RESULTS

### Patient population

From November 2013 to November 2014, 34 patients were enrolled. All patients underwent biopsy during radiology-guided or surgical procedures. Their characteristics are given in Table 1. Median age was 54 years (range, 35-77). Patients had TNBC (N=13, 32%), HER2-positive disease (N=7, 21%), or HR-positive/HER2-negative disease (N=14, 35%). Median disease-free interval was 49 months (range, 13-306), and 8 patients (24%) had synchronous metastases. A large number of patients had lymph node (N=19, 54%), liver (N=17, 50%), or bone (N=17, 50%) metastases at the time of enrolment. Most of patients had been heavily pre-treated by cytotoxics (N=33, 97%) including taxanes, anthracylines, alkylating agents and 5FU in 90% or more of the cases, endocrine therapies (N=23, 67%), or anti-HER2 drugs (N=7, 21%), according to specific subtypes. All patients but one had received at least one line of cytotoxic treatment in the adjuvant or advanced setting (median 3, range 1-9) and all but 4 had pretreatment for advanced stage, with a median number of 3 lines (range 1-8). Tumor biopsy sites were mainly liver and skin (Table 2) and median tumor cellularity was 70% (range, 10-90%). Two out of 34 patients had less than 30% of tumor cells and thus did not qualify for subsequent genomic analysis.

**Table 1:** Patient characteristics

	N = 34
**Age (years)**	
Median (range)	54 (35-77)
**Hormone receptors (HR) status**	
Positive	19 (55%)
Negative	15 (45%)
**HER2 status**	
Positive	7 (20%)
Negative	27 (80%)
**Tumor subtypes**	
Triple-negative	13 (38%)
HER2	7 (20%)
Luminal/HER2-negative	14 (42%)
**Metastases**	
Synchronous	8 (24%)
Metachronous	26 (76%)
**Disease-free interval (months)**	
Median (range)	49 (13-306)
< 24 months	9 (35%)
≥ 24 months	17 (65%)
**Previous treatment**	
***Cytotoxics (n, %)***	33 (97%)
Median number of lines (range)	3 (1-9)
Nature (n, % of treated)	
Anthracylines	30 (90%)
Taxanes	32 (97%)
5FU	30 (90%)
Eribuline	13 (39%)
Alkylating	30 (90%)
***Endocrine therapy (n, %)***	22 (35%)
Median number of lines (range)	2 (1-5)
Nature (n, % of treated)	
Tamoxifen	13 (59%)
Aromatase inhibitors	22 (100%)
Fulvestrant	3 (13%)
Everolimus-based	6 (27%)
***Anti-HER2 (n, %)***	7 (21%)
Median number of lines (range)	4 (1-6)
Nature (n, % of treated)	
Trastuzumab	7 (100%)
Lapatinib	3 (43%)
Trastuzumab emtansine	2 (29%)

**Table 2:** Tumor tissue

Tumor sites	N
Liver	15
Skin	6
Peritoneum	4
Breast	3
Lymph node	3
Lung	1
Pleura	1
Ascitis*	1

* Tumor cell pellet was obtained from ascites

### Molecular alterations and correlations with clinico-pathological features

**Figure 1:**
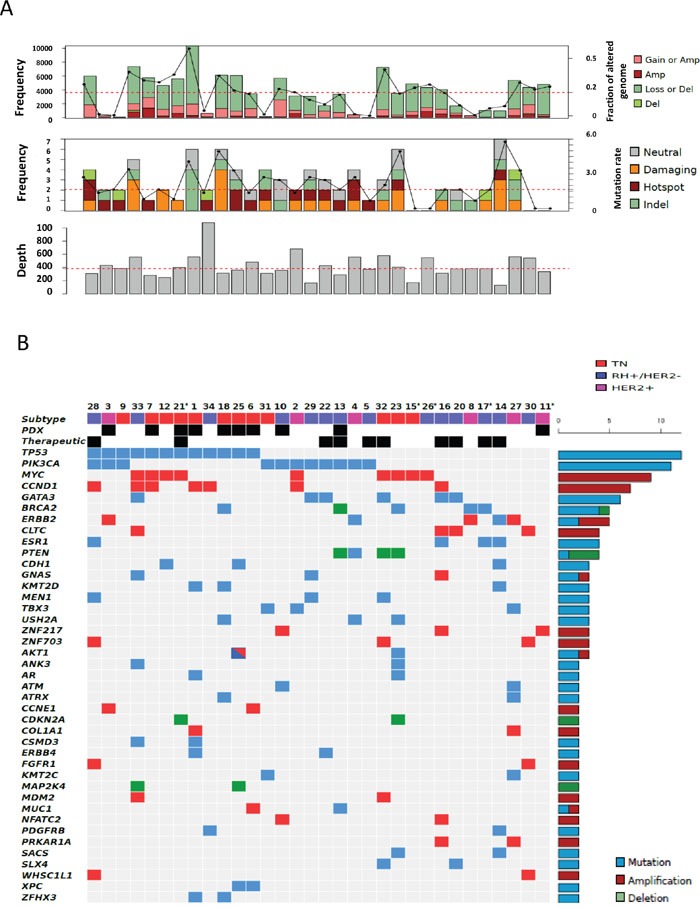
Distribution of molecular alterations identified by CGH arrays and NGS in all 32 samples **A.** Upper, mid and lower panels indicate the percentage of altered genomes, the mutation rate and the depth of sequencing for each sample, respectively (dot lines indicate mean percentage of altered genome, median mutation rate and median depth of sequencing, respectively). Gain, amplification, loss and deletion as well as neutral, damaging, hot spot and indel mutations were defined as indicated in the material and methods section. **B.** The distribution of molecular alterations (blue, mutation; red, amplification; green, deletion) was shown by decreasing frequency across samples (unique and anonymized patient number). Only alterations present in more than 1 sample are shown. Sample subtypes (HR-positive/HER2-negative, blue; HER2-positive, pink; triple-negative, red) are indicated. Patients with PDX engraftment (“PDX”) and genomic-driven treatment (therapeutic) are also shown. Asterisks indicate patients without germline sequencing.

**Figure 2:**
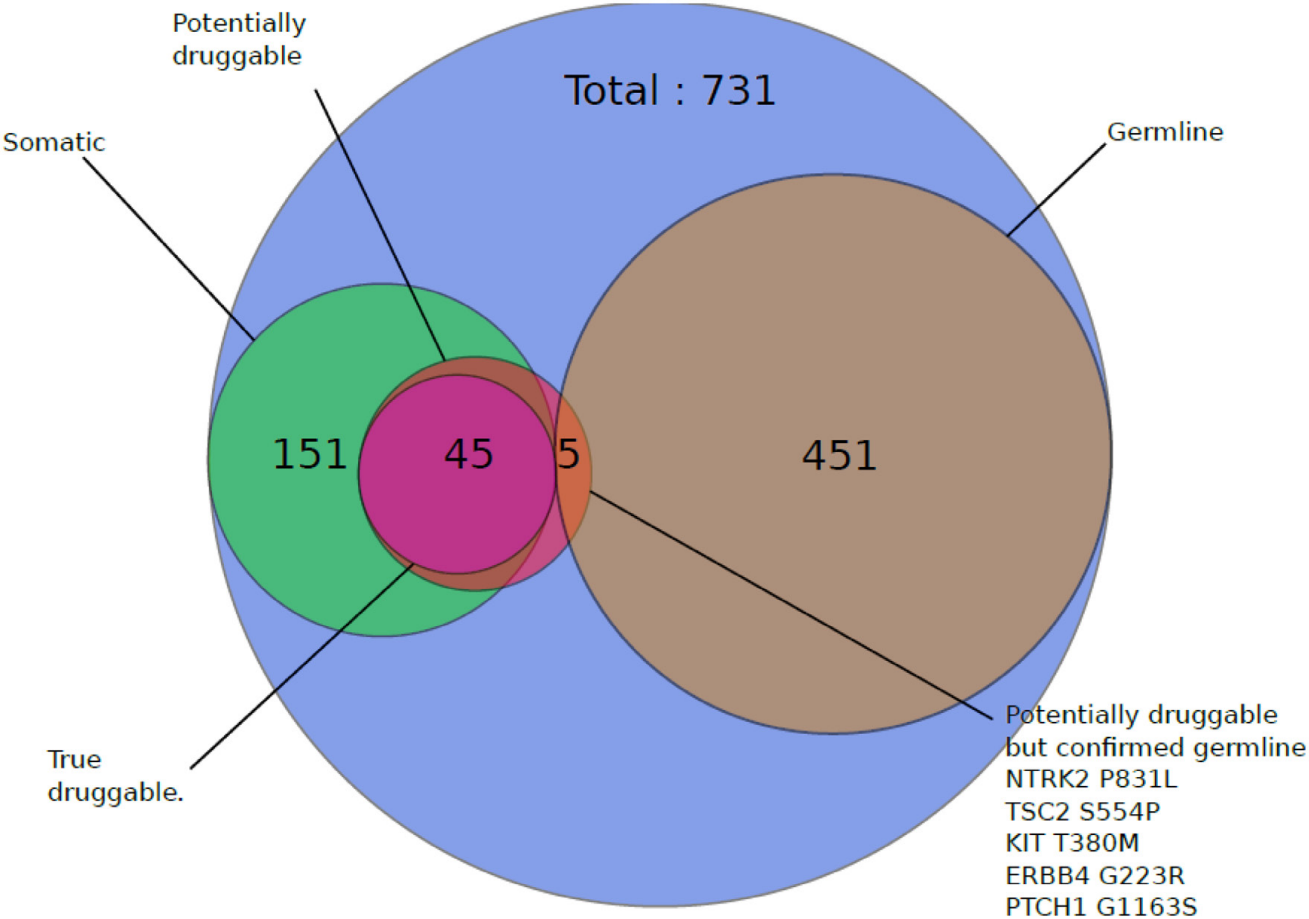
Impact of germline sequencing on the number of retained mutations Patients with available normal samples (N=27) were subjected to constitutional sequencing and somatic mutations were identified as indicated in the Material and methods section. Results are shown on a Venn diagram.

**Figure 3:**
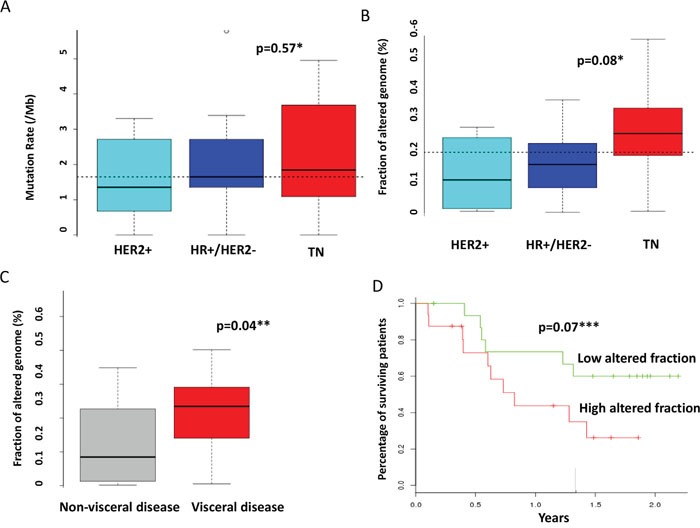
Correlations between molecular alterations and clinical features The mutation rate **A.** and the fraction of altered genome **B.** were calculated as indicated in the material and method section and compared across molecular subtypes. **C.** The fraction of altered genome was compared according to the presence of visceral or non-visceral metastases. **D.** Overall survival was compared according to the fraction of altered genome. The median value of the overall population (0.2) was selected as threshold. * ANOVA ** student's t-test *** Log-rank test.

Among the 32 patients evaluable for tumor NGS and aCGH (27 of which with constitutional sequencing available allowing identification of somatic variants), a total of 845 molecular alterations were identified, including 731 mutations, 95 amplifications, and 19 deletions (Figure 1). Of note, only 151 mutations were confirmed as somatic events after constitutional sequencing (Figure 2). The median rate of somatic mutations was 1.6 mutations per Mb and the mean percentage of altered genome was 19.5%. The mutation rate did not differ across subtypes (Figure 3A), but there was a non-significant trend toward a higher fraction of altered genome in triple-negative tumors (p=0.08, ANOVA; Figure 3B). A higher fraction of altered genome was associated with the presence of visceral metastases at the time of biopsy (p=0.04, Student t-test; Figure 3C) and a non-significant trend was observed for correlation with overall survival (p=0.07, Log-rank test, Figure 3D). Of note, there was no relationship between tumor-infiltrating lymphocytes (TILs), as evaluated on metastatic tissue, and mutation rate or fraction of altered genome. As expected, *TP53* mutations were associated with triple-negative subtype (8/12, *versus* 1/6 in HER2-positive and 3/14 in HR-positive/HER2-negative, p=0.039, Fisher's test).

### Actionable targets and molecular-driven therapeutics

An actionable target was found in 28 patients (82%, 95%CI 70.3-95.6%), either corresponding to a mutation (N=45), an amplification (N=20), or a deletion (N=7). Of note, constitutional sequencing affected the determination of actionable targets. Thus, seven potential mutations found in non-hotspot regions of actionable oncogenic drivers in seven patients were either validated (N=2) or invalidated (N=5) after constitutional analysis (Figure 2).

**Figure 4:**
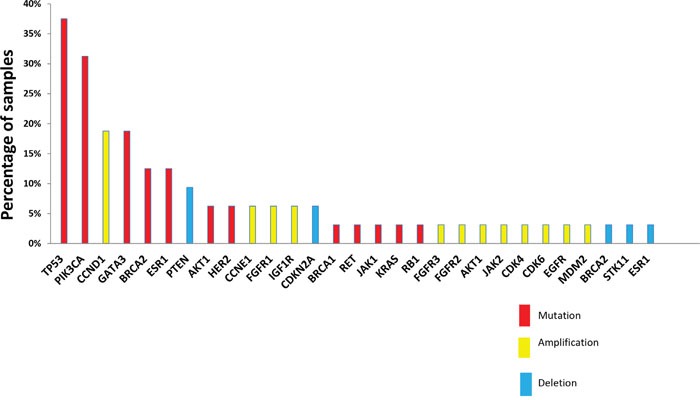
Actionable alterations retained by molecular tumor board Actionable alterations were selected according to rules defined in the material and methods section. Mutations, amplifications and deletions are shown across the samples analyzed (N=32) in red, yellow and blue, respectively.

The most frequent actionable alterations were *TP53* (12 of 32 patients analyzed, 37%), *PIK3CA* (10 patients, 31%; one additional *PIK3CA* somatic mutation was not retained as actionable since it did not involve kinase domain), *GATA3* (6 patients, 18%), *BRCA2* (4 patients, including 2 constitutional mutations, 12%), *ESR1* (4 patients, 12%) mutations, and *CCND1* amplifications (6 patients, 18%). Other actionable genomic alterations were identified in three or fewer cases (Figure 4).

Regarding the therapeutic applications (Table 3), at least one clinical trial evaluating a drug matching an identified molecular alteration was potentially available for 15 patients (44% of the 34 patients enrolled, 95%CI 28-60%), three of them being ultimately enrolled (based on *PIK3CA* mutation, *FGFR2* amplification, and *PTEN* deletion). No objective response was noted but two patients had stable disease with short duration. In 21 patients (61%, 95%CI 45-76%), a genomic-driven but off-trial therapeutic decision could be proposed. Off-trial treatment was actually delivered in 8 patients (including one patient which was subsequently enrolled in a clinical trial and was also mentioned in the paragraph above), based on *BRCA2* (carboplatin, N=1), *PIK3CA* (everolimus, N=4), *ESR1* (fulvestrant, N=2) mutations, and *JAK2* amplification (ruxolitinib, N=1). One partial response (carboplatin in patient with *BRCA2* mutation) and one long-lasting stable disease (exemestane-everolimus in patient with *PIK3CA* hot-spot mutation) were obtained. Thus, a total of ten patients (29%, 95%CI 13-44%) were treated based on genomics and two of them (i.e. 6% of the overall population) actually derived a clinical benefit from such a precision medicine strategy. Of note, genomic-driven treatments were more frequently delivered in HR-positive/HER2-negative (8 out of 14) than in other patients (2 out of 20; p=0.006, Fisher's test).

**Table 3:** Genomics-driven therapeutics

Clinical trial		
Number of patients eligible		15
Number of possible trial per patient (median, range)		1 (1-4)
Number of patients actually enrolled		3
***Targets***	***Targeted therapy***	***Best Response***
*PTEN* deletion	AKT/S6 inhibitor	Not available*
*PIK3CA* mutation	AKT/S6 inhibitor	Stable disease (≤4 months)**
*FGFR2* amplification	FGFR inhibiton	Stable disease (≤ 4 months)

“off-label” treatment proposal		
Number of patients eligible		21
Number of possible marketed therapy (median, range)		1 (1-3)
Number of patients treated		8
***Targets***	***Targeted therapy***	***Best Response***
*BRCA2* mut	carboplatin	Partial response
*PIK3CA* mut	everolimus	Stable disease (> 6 months)
*PIK3CA* mut	everolimus	Not available*
*PIK3CA* mut	everolimus	Progressive disease**
*PIK3CA* mut -*PTEN*del	everolimus	Progressive disease
*ESR1* mutation	fulvestrant	Progressive disease***
*ESR1* mutation	fulvestrant	Progressive disease
JAK2 amplification	ruxolitinib	Progressive disease

* Early treatment discontinuation due to toxicity

** This same patient was treated in both cohorts

*** This patient had dissociated response, with some lesions improved while other progressed

### Patient-derived xenograft (PDX) from metastatic tissue

Of 34 patients enrolled, 22 were subjected to xenotransplant. Grafting samples from the remaining patients was not attempted because of sample bacterial contamination, low amount of tumor material expected, or because the logistic of xenotransplant procedure did not fit the timing of the tumor biopsy. Of the 22 injected mice, 21 were monitored until engraftment or, in the absence of engraftment, for at least 18 months (for one PDX, all transplanted mice died within 1 month, presumably from infectious cause). Their characteristics are presented in Table 4. Ten PDX were successfully generated (29%, 95%CI 16-46% of the overall population; 45%, 95%CI 26-65% of patients subjected to engraftment). Successful PDXs were most frequently from triple-negative tumors (N=6, out of 8 attempted), rather than from other subtypes: 2 out of 5 HER2-positive (40%) and 2 out of 8 HR-positive/HER2-negative (25%) xenotransplanted samples. Median time to reach 10 mm was 176 days (range 50-419) and was also subtype-dependent: 99 (range, 50-132) and 294 (range, 182-419) days in triple-negative and non-triple-negative tumors, respectively (p=0.009, Mann-Whitney's test). The most frequent site of tumor biopsy in successful PDXs was liver (N=4). Metastatic disease was metachronous in 8 patients and synchronous in 2 patients. Of note, patients with successful PDX had a trend for shorter disease-free interval than had patients whose tumor engraftment failed (22 *versus* 95 months; p=0.07, Mann-Whitney's test).

**Table 4:** Patients subjected to xenograft: clinical and pathological features

Pt number	Age	(p)TNM	SBR grade	Vascular invasion	Biopsy site	Subtype	Disease sites	disease-free interval (months)	Prior chemoth.	Engraftment (days)
1	57	pT2N0M0	3	POS	LUNG	TN	BRAIN, NODE, LUNG, LIVER	26	4	YES (82)
3	65	T4dN2M0	3	UK	SKIN	HER2+	SKIN	13	2	YES (182)
4	73	pT1N0M1	2	NEG	PERITONEUM	HER2+	BONE, PERITONEUM, UTERUS, NODE	103	6	NO
6	49	T3N1M1	3	UK	BREAST	TN	SKIN, BREAST	NA	5	YES (132)
7	45	pT2N1M0	3	POS	LIVER	TN	LIVER, NODE, BONE	14	1	YES (50)
8	58	pTxNxM0	UK	UK	PERITONEUM	HER2+	PERITONEUM	51	1	NO
9	48	T1N1M1	UK	UK	LIVER	TN	LIVER, BONE	NA	2	NO
10	41	pT2N1bM0	3	POS	LIVER	RH+/HER2-	LIVER, BONE	14	9	YES (419)
11	69	pT1N0M0	2	UK	LIVER	HER2+	LIVER	85	8	YES (329)
12	51	T4dN2M0	3	NA	SKIN	TN	SKIN, NODES, LUNG, PLEURA	17	2	NO
13	68	pT1N1M0	2	POS	BREAST	RH+/HER2-	BONE, NODE LIVER, BREAST	171	1	YES (259)
14	71	pT2N2M0	UK	UK	PERITONEUM	RH+/HER2-	BONE, PERITONEUM	134	2	NO
16	54	pT2N0M0	3	NEG	LIVER	RH+/HER2-	LIVER, BONE NODE	95	6	NO
17	56	pTxNxM0	UK	UK	PERITONEUM	RH+/HER2-	PERITONEUM, LIVER	100	4	NO
18	42	TxNxM0	UK	UK	SKIN	TN	SKIN, NODES PANCREAS	20	3	YES (116)
19	54	TxN1M0	UK	UK	SKIN	HER2+	SKIN, NODES	72	4	NO
20	37	pT1N1M0	3	NEG	LIVER	RH+/HER2-	LIVER, BONE	24	4	NO
21	52	pT1N0M0	3	POS	LIVER	TN	NODES, LIVER	23	4	YES (131)
22	43	T0N3M1	2	NEG	LIVER	RH+/HER2-	LUNG, BONE, NODE, LIVER	NA	2	NO
25	64	T0N1M1	3	UK	ASCITIS	TN	PERITONEUM, NODES	NA	3	YES (51)
26	78	pTxNxM0	UK	UK	SKIN	RH+/HER2-	SKIN, BONE	119	2	NO

* Only patients transplanted and evaluable for engraftment are shown (N=21)

## DISCUSSION

In this pilot study we have reported the prospective implementation of precision medicine for aBC patients in a single center, using targeted NGS- and aCGH-based genomic analysis. We have found a high number of patients with actionable targets (82%), some of them driving an actual therapeutic decision (in 10 patients, i.e. 29% of the overall population) and possibly a clinical benefit (in 2 patients, i.e. 6% of the overall population). Moreover, for the first time to our knowledge, we have prospectively evaluated the ability to derive PDX from metastatic BC tissue, in the context of a prospective clinical study. Nearly 30% of patients (and more than 45% in the population in which xenotransplant was attempted), mainly triple-negative aBC, had a successful PDX.

A recent, large, French, multicentric prospective study (SAFIR 01) evaluating the search for hot-spot mutations of *AKT1* and *PIK3CA*, coupled to aCGH for copy number alterations, in more than 400 advanced breast cancer patients, demonstrated the feasibility of obtaining biopsy from metastatic tissue for molecular analyses [14]. Also consistent with our results, this study found a large number of relatively rare molecular alterations and a high level of patients with targetable targets (46%). The even higher percentage found in the present study, which may be due to the higher number of genes analyzed using targeted-NGS, logically translated in a larger number of patients (10 of 34, 29% *versus* 13%) actually receiving a genomic-oriented treatment. However, in both studies, only a low number of patients were actually directed toward a specific clinical trial and the actual number of patients deriving a clinical benefit was low (less than 10% of the initial population). Thus, whether molecular screening for precision medicine should use large panels of genes rather than selected targets (known to be actionable and with available corresponding therapeutics) remains to be investigated. It is not certain that extending the genomic analysis to full exomes or even to whole genomes (which may be associated with lower depth) could provide more truly actionable targets in the absence of more available drugs.

Other important analytic issues of potential critical relevance for genomic-based approaches include the required depth of sequencing, the need for tools allowing discriminating between passenger alterations and actual oncogenic drivers, as well as the requirement for simultaneous constitutional sequencing to validate the relevance of identified molecular alterations. In our study, the latter procedure had an impact on the identification of actionable targets, since several non-hotspot mutations in possible oncogenic drivers (5 of 50 mutations, i.e. 10%) were not confirmed by constitutional analysis, affecting the potential targeted treatment of 7 patients (20% of the total population). Another important point is the relative performance of tissue *versus* blood sequencing. Indeed, the fast development of robust genomic analyses on plasma-derived cell-free DNA could allow a more dynamic molecular typing [18]. In addition, it could reflect more effectively the intratumor heterogeneity, which was recently considered as an important limitation for precision medicine [19]. Indeed, molecular alterations detected by genomic profiling of tumor biopsy may not be shared by the majority of tumor cells of the biopsied site as well as by other metastatic deposits, which may significantly impede the antitumor activity of tested targeted therapies.

Consistent with these relatively disappointing results, SHIVA trial, the first randomized clinical trial ever performed evaluating the concept of precision medicine, did not demonstrate any survival advantage for the use of registered targeted treatment matching genomic alterations compared to treatment at physician's choice in various advanced solid tumors including breast cancer [20]. Such a low antitumor activity observed in precision medicine strategies, including the present study, may be due to the long, complex and treatment-rich pretreatment history of the disease, the suboptimal efficacy of offered targeted therapies at least as single-agent, rather than to the poor predictive value of detected molecular alterations, predictive value that however might be improved with multigene scores. Another critical limitation to routine implementation of precision medicine strategies remains the relatively low access to molecularly-targeted innovative clinical trials, which are mostly developed in high-volume comprehensive cancer centers, due to the expected low prevalence of the target population. Treating patients with less advanced-stage disease and using more innovative agents is currently tested in the randomized phase 2 trial SAFIR02 (NCT02299999) in which genomics-matched, targeted treatments are used as maintenance after response or stabilization obtained by standard first- or second-line cytotoxic chemotherapy in HER2-negative advanced breast cancer patients. Other major points to be incorporated in the design of future clinical trials include the standardization of experimental techniques to be used and their possible adaptation during the study, the use of bioinformatics-guided multidimensional algorithm [21], integrating coexisting molecular alterations, allowing prioritizing and selecting the best therapeutic options [22], the use of additional predictors such as functional assays, circulating tumor cells or cell-free DNA and the corresponding use of combinatorial therapeutics, including association of targeted therapies and/or immuno-oncology agents.

A possible way for improving efficacy of precision medicine may rely upon complementing genomic data with functional assays, in which available therapeutics are tested on preclinical models before being administered to patients. As demonstrated in our study, PDXs could be generated with a significant rate of success, notably in triple-negative patients, a subtype in which targeted therapies are desperately lacking. Notably, the rate of engraftment metastasis-derived tissue (45%) was higher than we previously reported (27%) for primary breast cancer [17]. Even though the time to engraftment remains a limiting factor for directing immediate treatment according to PDX-based preclinical evaluation, these tumor avatars might be helpful to guide or adjust subsequent lines of treatment. Thus, if biopsy is obtained immediately at the time of metastasis diagnosis, empirically-selected conventional first-line treatment may be proposed, while PDX is growing and then phenotypically characterized. Of note, the median time for PDX engraftment observed in our study remains inferior to the median progression-free survival of standard first-line treatments for metastatic breast cancer, suggesting that results of *in-vivo* preclinical evaluation might be actually usable to direct either maintenance treatment in responding patients or rescue strategies for progressive disease. In addition, novel *ex-vivo* preclinical models such as 3D-organoïds and/or short-term cell cultures are emerging [23, 24], that may shorten delay for phenotyping experiments and be helpful in case of graft failure. Whether these preclinical models may improve efficacy of precision medicine should be evaluated in a prospective study, the feasibility of which is supported by our data. A putative clinical trial testing this hypothesis is provided as Supplementary Figure S1.

Interestingly, and somewhat counterintuitively, there was no association between molecular subtypes and mutation rates; in particular triple-negative tumors did not have significantly more mutations than other subtypes. However, an increased fraction of altered genome was associated with triple-negative subtype, visceral involvement and reduced overall survival. It has been hypothesized that the level of genomic alterations, including the so-called mutational load, may favor the emergence of neo-antigens and thus may stimulate immune response associated with improved outcome and/or efficacy of immunotherapy [25–27]. Yet, in the present study, the TILs percentage did not correlate with the mutation rate or the fraction of altered genomes. Whether this observation is due to small sample size, technical imprecision of TILs enumeration on small biopsy, or actual inability of highly mutated metastatic disease to recruit immune effectors remains to be determined. Correlations between specific molecular alterations and survival were also exploratory investigated and we observed an association between mutations of *PIK3CA* (N=11) and a better outcome (HR=0.28, 95%CI, 0.08-0.98; p=0.046, Log-rank test), whereas *AKT1* mutations (N=2) correlated with an increased risk of death (HR=21.21, 95%CI, 1.27-353.59; p=0.033, Log-rank test). However, regarding to the very limited sample size, these data require further validation on larger and independent samples.

In conclusion, our pilot study shows that precision medicine may be implemented prospectively in a single-center, but with a limited clinical benefit at this point and with this selection of advanced cases. Thus, our data are consistent with previous studies, in terms of potential and current limitations of precision medicine. However, this is the first study also demonstrating the ability of a prospective precision medicine strategy to derive in a significant percentage of cases *in vivo* preclinical models that have the potential for improving efficacy of therapeutic management. Finally, molecular examination of advanced forms of disease may reveal some relevant associations with clinical features and outcome.

## MATERIALS AND METHODS

### Patients and samples

Between November, 2013 and November, 2014, 34 patients with metastatic or locally- advanced breast cancer accessible to tumor biopsy were prospectively enrolled at the Institut Paoli-Calmettes in the BC-BIO study (ClinicalTrials.gov, NCT01521676). This study is an ongoing institutional clinical study dedicated to molecular characterization of breast cancer, which was approved by our local ethics committee. After giving their informed consent for translational research, including genetic analyses of their germline DNA, tumor biopsy was obtained by any visually- or radiology-guided percutaneous or surgical biopsy. Samples were divided in 3 parts. A first part was fixed and paraffin-embedded for diagnosis and standard immunohistochemistry (IHC). A second part was frozen after control for cellularity and only samples with at least 30% of tumor cells were retained for subsequent genomic analyses. A third part was dissociated mechanically and enzymatically using collagenase/hyaluronidase digestion to generate single-cell suspension for *in vivo* implantation in mice. Formalin-fixed paraffin-embedded (FFPE) samples were analyzed for estrogen receptor (ER), progesterone receptor (PR), and HER2 expression using standard guidelines [28, 29]. Tumor-infiltrating lymphocytes (TILs) were morphologically evaluated on FFPE samples, by examination of hematoxylin and eosin (H&E)-stained tumor sections, as recommended [30]. The following clinical and biological items were recorded: date of birth, sex, clinico-pathological features of primary breast cancer (date of diagnosis, stage, SBR grade, perivascular invasion, ER, PR, and HER2 statutes, adjuvant/neoadjuvant chemotherapy, radiotherapy, endocrine therapy, trastuzumab) and of locally-advanced or metastatic relapse (date of diagnosis, disease-free interval, nature and number of lines of cytotoxic, endocrine and/or targeted treatments before biopsy), sites of metastatic disease, site and date of tumor biopsy, cellularity, and date of death or date of last news following biopsy.

### Array-comparative genomic hybridization (aCGH)

Tumor DNAs were extracted as previously described [31] and controlled on Agilent Bioanalyzer (Agilent Technologies, Massy, France). Genomic profiles of 32 samples were established by using aCGH onto high-resolution 4×180K CGH microarrays (SurePrint G3 Human CGH Microarray Kit, Agilent Technologies, Massy, France). Human female DNA was used as reference (G152A, Promega). Both approaches and analysis methods are described in previous studies [32–34]. All probes for aCGH were mapped according to the hg19/NCBI human genome mapping database. The copy-number was estimated for each gene by taking the value of the segment with the highest amplitude, then categorized into “Amp” (Log2ratio > 1), “Gain” (0.5 < Log2ratio <= 1), “Loss” (−1 <= Log2ratio < −0.3) and “Del” (Log2ratio <-1). Focal events were defined as genomic alterations with a size less than 5 Mb and a copy number higher than the surrounding segments. Percentage of genome altered was calculated as the sum of altered probes divided by the total number of probes.

### Next-generation sequencing (NGS)

Targeted NGS was applied to a custom-made panel of 365 “cancer-associated” genes selected for their involvement in cancers (CCP-V6 panel, previously described in [34]). For 32 tumor samples, we prepared the DNA libraries of all coding exons and intron-exon boundaries of all genes using the HaloPlex Target Enrichment System (Agilent, Santa Clara, CA, USA) as described [35]. Sequencing was done using the 2×150-bp paired-end technology on the Illumina MiSeq platform according to the manufacturer's instructions (Illumina, San Diego, CA, USA). In order to identify only somatic mutations, germline DNAs were similarly sequenced for 27 normal counterpart samples (blood lymphocytes of corresponding patients). Five normal samples were missing.

### Bioinformatics processing

In a first data analysis pipeline, tumor sequence data were aligned to the human reference genome (UCSC hg19) using Burrows-Wheeler Aligner [36]. Samples were sequenced at an average depth of 380x for the targeted regions. Bam files were processed as described [35]. Then, the single nucleotide variants (SNVs) calling was done with FreeBayes version 0.9.9 [37] with a minimal alternate variant frequency and coverage set at 0.02 and 10. Insertions/deletions (indels) calling was done using GATK haplotype caller version 2.5-2-gf57256b [38] with default parameters. The variants, i.e. SNVs and indels, were annotated with the Annotate Variation Software (ANNOVAR, version 2013-11-12). Known variants found in dbsnp129 and dbsnp137 with a variant allele frequency (VAF) superior to 1% (1000G or ESP6500) were removed. Finally, low frequency SNVs and indels that were suspected to be false positive were systematically inspected with IGV version 2.3.32 [39, 40]. In a second data analysis pipeline, both tumor and germinal sequences were analyzed using the same procedure with light modifications. Somatic SNV calling was done with Mutect 1.7 [41] and somatic indel calling was done with Scalpel. All variants were then annotated for genes and function using ANNOVAR. Exonic (non-synonymous, stopgain, stoploss, ins/del frameshift, ins/del non-frameshift) and splicing variants found somatic were kept. In order to remove false positives, recurrent variants with none entry in public databases such as COSMIC or dbsnp were removed. In addition, SNV with a t_lod_fstar score under 80 or with an alternate allele frequency < 5% as well as the indels were systematically inspected with IGV. At last, variants identified by both pipeline analyses were retained as somatic. The mutation rate was calculated as the sum of identified mutations divided by the sequenced region size.

### Patient-derived xenograft (PDX) generation

Tumor biopsy was subjected to mechanic and enzymatic dissociation using collagenase/hyaluronidase (StemCell Technologies) digestion, and a single-cell suspension was obtained and used for the *in vivo* implantation. For each biopsy, 1 × 10^6^ cells were implanted in humanized cleared fat pads of 3 NSG (NOD/Shi-scid/IL-2Rγnull) mice and engraftment was monitored as previously described [17].

### Molecular tumor board

Results from genomic analyses were discussed by our local molecular tumor board (MTB), including Medical Oncologists (An. G, FB), Pathologists (ECJ, JTP), Medical Geneticists (CP, FE, HS), Bio-informatics scientists (Ar. G, PF) and Biologists (JA, ML, CG, MC, DB). Possibly actionable molecular targets were consensually defined, as follows: only focal amplifications and/or hotspot activating mutations in known oncogenic drivers, as well as homozygous deletion or heterozygous deletion associated with inactivating mutation of tumor suppressor genes. Possibly activating mutations in non-hot spot regions of oncogenic drivers were also retained when confirmed as somatic by comparison with sequencing of normal tissue. Targeted therapeutic propositions were made according to molecular targets identified as proposed in [42], specific clinical trials available at our institution or other centers in France, and already registered targeted therapies. Patients actually receiving treatment according to MTB proposal were monitored for tumor response.

### Statistical analysis

Comparisons of categorical variables distribution were performed by χ2 or Fisher's exact test. For continuous variables, ANOVA, Student's t-test or non-parametric Mann-Whitney U test were used. Follow-up was calculated from the date of biopsy to the date of last news for patients alive. Overall survival (OS) was measured from biopsy until death or date of last news, estimated with the Kaplan–Meier method and compared between groups with the log-rank test. All statistical tests were two-sided. Statistical analyses were performed either with the R software version 2.15 or Prism software (Graphpad software, San Diego, CA, USA).

## SUPPLEMENTARY FIGURE



## References

[1] Ferlay J, Soerjomataram I, Dikshit R, Eser S, Mathers C, Rebelo M, Parkin DM, Forman D, Bray F (2015). Cancer incidence and mortality worldwide: sources, methods and major patterns in GLOBOCAN 2012. Int J Cancer J Int Cancer.

[2] Jemal A, Siegel R, Ward E, Hao Y, Xu J, Murray T (2008). Cancer statistics, 2008. CA Cancer J Clin.

[3] Joy AA, Ghosh M, Fernandes R, Clemons MJ (2015). Systemic treatment approaches in her2-negative advanced breast cancer—guidance on the guidelines. Curr Oncol.

[4] Perou C, Sorlie T, Eisen M, van de Rijn M, Jeffrey S, Rees C, Pollack J, Ross D, Johnsen H, Akslen L (2000). Molecular portraits of human breast tumours. Nature.

[5] Network TCGA (2012). Comprehensive molecular portraits of human breast tumours. Nature.

[6] Nik-Zainal S, Davies H, Staaf J, Ramakrishna M, Glodzik D, Zou X, Martincorena I, Alexandrov LB, Martin S, Wedge DC, Van Loo P, Ju YS, Smid M (2016). Landscape of somatic mutations in 560 breast cancer whole-genome sequences. Nature.

[7] Garraway LA, Verweij J, Ballman KV (2013). Precision oncology: an overview. J Clin Oncol.

[8] Early Breast Cancer Trialists' Collaborative Group (EBCTCG) (2005). Effects of chemotherapy and hormonal therapy for early breast cancer on recurrence and 15-year survival: an overview of the randomised trials. Lancet.

[9] Slamon DJ, Leyland-Jones B, Shak S, Fuchs H, Paton V, Bajamonde A, Fleming T, Eiermann W, Wolter J, Pegram M, Baselga J, Norton L (2001). Use of chemotherapy plus a monoclonal antibody against HER2 for metastatic breast cancer that overexpresses HER2. N Engl J Med.

[10] Romond EH, Perez EA, Bryant J, Suman VJ, Geyer CE, Davidson NE, Tan-Chiu E, Martino S, Paik S, Kaufman PA, Swain SM, Pisansky TM, Fehrenbacher L (2005). Trastuzumab plus adjuvant chemotherapy for operable HER2-positive breast cancer. N Engl J Med.

[11] Piccart-Gebhart MJ, Procter M, Leyland-Jones B, Goldhirsch A, Untch M, Smith I, Gianni L, Baselga J, Bell R, Jackisch C, Cameron D, Dowsett M, Barrios CH (2005). Trastuzumab after adjuvant chemotherapy in HER2-positive breast cancer. N Engl J Med.

[12] Stephens PJ, Tarpey PS, Davies H, Van Loo P, Greenman C, Wedge DC, Nik-Zainal S, Martin S, Varela I, Bignell GR, Yates LR, Papaemmanuil E, Beare D (2012). The landscape of cancer genes and mutational processes in breast cancer. Nature.

[13] Curtis C, Shah SP, Chin S-F, Turashvili G, Rueda OM, Dunning MJ, Speed D, Lynch AG, Samarajiwa S, Yuan Y, Gräf S, Ha G, Haffari G (2012). The genomic and transcriptomic architecture of 2,000 breast tumours reveals novel subgroups. Nature.

[14] André F, Bachelot T, Commo F, Campone M, Arnedos M, Dieras V, Lacroix-Triki M, Lacroix L, Cohen P, Gentien D, Adélaide J, Dalenc F, Gonçalves A (2014). Comparative genomic hybridisation array and DNA sequencing to direct treatment of metastatic breast cancer: a multicentre, prospective trial (SAFIR01/UNICANCER). Lancet Oncol.

[15] DeRose YS, Wang G, Lin Y-C, Bernard PS, Buys SS, Ebbert MTW, Factor R, Matsen C, Milash BA, Nelson E, Neumayer L, Randall RL, Stijleman IJ (2011). Tumor grafts derived from women with breast cancer authentically reflect tumor pathology, growth, metastasis and disease outcomes. Nat Med.

[16] Zhang X, Claerhout S, Prat A, Dobrolecki LE, Petrovic I, Lai Q, Landis MD, Wiechmann L, Schiff R, Giuliano M, Wong H, Fuqua SW, Contreras A (2013). A renewable tissue resource of phenotypically stable, biologically and ethnically diverse, patient-derived human breast cancer xenograft models. Cancer Res.

[17] Charafe-Jauffret E, Ginestier C, Bertucci F, Cabaud O, Wicinski J, Finetti P, Josselin E, Adelaide J, Nguyen T-T, Monville F, Jacquemier J, Thomassin-Piana J, Pinna G (2013). ALDH1-positive cancer stem cells predict engraftment of primary breast tumors and are governed by a common stem cell program. Cancer Res.

[18] De Mattos-Arruda L, Caldas C (2015). Cell-free circulating tumour DNA as a liquid biopsy in breast cancer. Mol Oncol.

[19] Jamal-Hanjani M, Quezada SA, Larkin J, Swanton C (2015). Translational implications of tumor heterogeneity. Clin Cancer Res.

[20] Le Tourneau C, Delord J-P, Gonçalves A, Gavoille C, Dubot C, Isambert N, Campone M, Trédan O, Massiani M-A, Mauborgne C, Armanet S, Servant N, Bièche I (2015). Molecularly targeted therapy based on tumour molecular profiling versus conventional therapy for advanced cancer (SHIVA): a multicentre, open-label, proof-of-concept, randomised, controlled phase 2 trial. Lancet Oncol.

[21] Le Tourneau C, Kamal M, Tsimberidou A-M, Bedard P, Pierron G, Callens C, Rouleau E, Vincent-Salomon A, Servant N, Alt M, Rouzier R, Paoletti X, Delattre O (2016). Treatment Algorithms Based on Tumor Molecular Profiling: The Essence of Precision Medicine Trials. J Natl Cancer Inst.

[22] Swanton C, Soria J-C, Bardelli A, Biankin A, Caldas C, Chandarlapaty S, de Koning L, Dive C, Feunteun J, Leung S-Y, Marais R, Mardis ER, McGranahan N (2016). Consensus on precision medicine for metastatic cancers: a report from the MAP conference. Ann Oncol.

[23] Bosserman L, Rogers K, Willis C, Davidson D, Whitworth P, Karimi M, Upadhyaya G, Rutledge J, Hallquist A, Perree M, Presant CA (2015). Application of a drug-induced apoptosis assay to identify treatment strategies in recurrent or metastatic breast cancer. PloS One.

[24] Francies HE, Barthorpe A, McLaren-Douglas A, Barendt WJ, Garnett MJ (2016). Drug Sensitivity Assays of Human Cancer Organoid Cultures. Methods Mol Biol.

[25] Rooney MS, Shukla SA, Wu CJ, Getz G, Hacohen N (2015). Molecular and genetic properties of tumors associated with local immune cytolytic activity. Cell.

[26] Brown SD, Warren RL, Gibb EA, Martin SD, Spinelli JJ, Nelson BH, Holt RA (2014). Neo-antigens predicted by tumor genome meta-analysis correlate with increased patient survival. Genome Res.

[27] Howitt BE, Shukla SA, Sholl LM, Ritterhouse LL, Watkins JC, Rodig S, Stover E, Strickland KC, D'Andrea AD, Wu CJ, Matulonis UA, Konstantinopoulos PA (2015). Association of Polymerase e-Mutated and Microsatellite-Instable Endometrial Cancers With Neoantigen Load, Number of Tumor-Infiltrating Lymphocytes, and Expression of PD-1 and PD-L1. JAMA Oncol.

[28] Hammond MEH, Hayes DF, Wolff AC, Mangu PB, Temin S (2010). American society of clinical oncology/college of american pathologists guideline recommendations for immunohistochemical testing of estrogen and progesterone receptors in breast cancer. J Oncol Pract Am Soc Clin Oncol.

[29] Wolff AC, Hammond MEH, Hicks DG, Dowsett M, McShane LM, Allison KH, Allred DC, Bartlett JMS, Bilous M, Fitzgibbons P, Hanna W, Jenkins RB, Mangu PB (2013). Recommendations for human epidermal growth factor receptor 2 testing in breast cancer: American Society of Clinical Oncology/College of American Pathologists clinical practice guideline update. J Clin Oncol.

[30] Desmedt C, Di Leo A, de Azambuja E, Larsimont D, Haibe-Kains B, Selleslags J, Delaloge S, Duhem C, Kains J-P, Carly B, Maerevoet M, Vindevoghel A, Rouas G (2011). Multifactorial approach to predicting resistance to anthracyclines. J Clin Oncol.

[31] Adelaide J, Finetti P, Bekhouche I, Repellini L, Geneix J, Sircoulomb F, Charafe-Jauffret E, Cervera N, Desplans J, Parzy D, Schoenmakers E, Viens P, Jacquemier J (2007). Integrated profiling of basal and luminal breast cancers. Cancer Res.

[32] Sircoulomb F, Bekhouche I, Finetti P, Adélaïde J, Ben Hamida A, Bonansea J, Raynaud S, Innocenti C, Charafe-Jauffret E, Tarpin C, Ben Ayed F, Viens P, Jacquemier J (2010). Genome profiling of ERBB2-amplified breast cancers. BMC Cancer.

[33] Bekhouche I, Finetti P, Adelaïde J, Ferrari A, Tarpin C, Charafe-Jauffret E, Charpin C, Houvenaeghel G, Jacquemier J, Bidaut G, Birnbaum D, Viens P, Chaffanet M (2011). High-resolution comparative genomic hybridization of inflammatory breast cancer and identification of candidate genes. PloS One.

[34] Bertucci F, Finetti P, Guille A, Adélaïde J, Garnier S, Carbuccia N, Monneur A, Charafe-Jauffret E, Gonçalves A, Viens P, Birnbaum D, Chaffanet M (2016). Comparative genomic analysis of primary tumors and metastases in breast cancer. Oncotarget.

[35] Collette Y, Prébet T, Goubard A, Adélaïde J, Castellano R, Carbuccia N, Garnier S, Guille A, Arnoulet C, Charbonier A, Mozziconacci MJ, Birnbaum D, Chaffanet M (2015). Drug response profiling can predict response to ponatinib in a patient with t(1;9)(q24;q34)-associated B-cell acute lymphoblastic leukemia. Blood Cancer J.

[36] Li H, Durbin R (2009). Fast and accurate short read alignment with Burrows-Wheeler transform. Bioinforma Oxf Engl.

[37] Garrison E, Marth G (2012). Haplotype-based variant detection from short-read sequencing. ArXiv12073907 Q-Bio.

[38] DePristo MA, Banks E, Poplin R, Garimella KV, Maguire JR, Hartl C, Philippakis AA, del Angel G, Rivas MA, Hanna M, McKenna A, Fennell TJ, Kernytsky AM (2011). A framework for variation discovery and genotyping using next-generation DNA sequencing data. Nat Genet.

[39] Robinson JT, Thorvaldsdóttir H, Winckler W, Guttman M, Lander ES, Getz G, Mesirov JP (2011). Integrative genomics viewer. Nat Biotechnol.

[40] Thorvaldsdóttir H, Robinson JT, Mesirov JP (2013). Integrative Genomics Viewer (IGV): high-performance genomics data visualization and exploration. Brief Bioinform.

[41] Cibulskis K, Lawrence MS, Carter SL, Sivachenko A, Jaffe D, Sougnez C, Gabriel S, Meyerson M, Lander ES, Getz G (2013). Sensitive detection of somatic point mutations in impure and heterogeneous cancer samples. Nat Biotechnol.

[42] Dienstmann R, Jang IS, Bot B, Friend S, Guinney J (2015). Database of genomic biomarkers for cancer drugs and clinical targetability in solid tumors. Cancer Discov.

