# Supplementary material for: Targeted NGS, array-CGH, and patient-derived tumor xenografts for precision medicine in advanced breast cancer: a single-center prospective study
Source: Oncotarget. 2016 Oct 18;7(48):79428–41. doi: 10.18632/oncotarget.12714 (PMC5346725; doi:10.18632/oncotarget.12714)
Supplement: Supplementary file 1 [file oncotarget-07-79428-s001.pdf]

Targeted NGS, array-CGH, and patient-derived tumor xenografts for precision medicine in advanced breast cancer: a single-center prospective study

SUPPLEMENTARY FIGURE

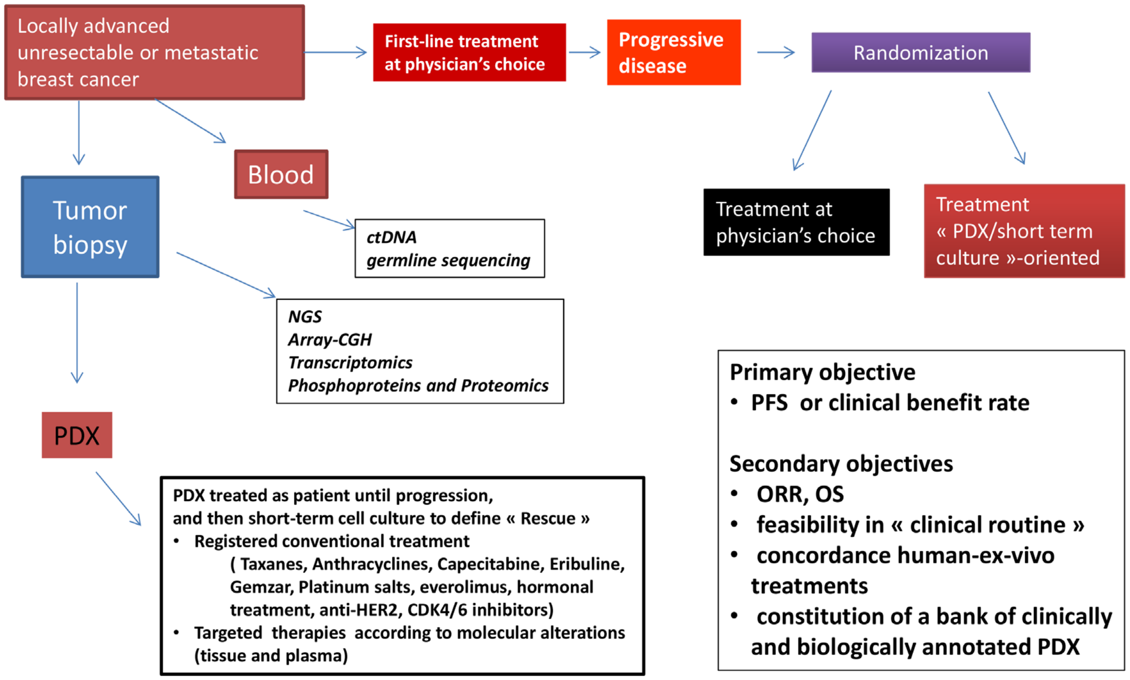

Supplementary Figure S1: A putative clinical trial testing treatment according to *in-vivo* phenotyping *versus* empirical treatment. PDX: patient-derived xenograft, ctDNA: circulating tumor DNA, PFS: progression-free survival, ORR: overall response rate, OS: overall survival.
